# Supplementary material for: Modeling glioblastoma heterogeneity as a dynamic network of cell states
Source: Mol Syst Biol. 2021 Sep 16;17(9):e10105. doi: 10.15252/msb.202010105 (PMC8444284; doi:10.15252/msb.202010105)
Supplement: Supplementary file 6 — Source Data for Figure 5 [file MSB-17-e10105-s004.zip › Figure5A_sourcedata/GSEA_3017/hallmarks_stateE.GseaPreranked.1621934677158/HALLMARK_MITOTIC_SPINDLE.html]

Details for gene set HALLMARK\_MITOTIC\_SPINDLE[GSEA]

|  || Dataset | state63017 |
| Phenotype | NoPhenotypeAvailable |
| Upregulated in class | na\_neg |
| GeneSet | HALLMARK\_MITOTIC\_SPINDLE |
| Enrichment Score (ES) | -0.58406794 |
| Normalized Enrichment Score (NES) | -2.796495 |
| Nominal p-value | 0.0 |
| FDR q-value | 0.0 |
| FWER p-Value | 0.0 |
Table: GSEA Results Summary

  

Fig 1: Enrichment plot: HALLMARK\_MITOTIC\_SPINDLE      
 Profile of the Running ES Score & Positions of GeneSet Members on the Rank Ordered List

  

| PROBE | GENE SYMBOL | GENE\_TITLE | RANK IN GENE LIST | RANK METRIC SCORE | RUNNING ES | CORE ENRICHMENT || 1 | NEDD9 |  |  | 38 | 0.590 | -0.0257 | No |
| 2 | EZR |  |  | 96 | 0.410 | -0.0779 | No |
| 3 | ARHGAP29 |  |  | 175 | 0.314 | -0.1564 | No |
| 4 | SPTBN1 |  |  | 194 | 0.300 | -0.1680 | No |
| 5 | FLNA |  |  | 197 | 0.298 | -0.1616 | No |
| 6 | KIFAP3 |  |  | 208 | 0.290 | -0.1645 | No |
| 7 | SORBS2 |  |  | 249 | 0.262 | -0.2019 | No |
| 8 | EPB41 |  |  | 345 | -0.268 | -0.3008 | No |
| 9 | KNTC1 |  |  | 352 | -0.269 | -0.2998 | No |
| 10 | CNTRL |  |  | 462 | -0.304 | -0.4135 | No |
| 11 | SMC1A |  |  | 472 | -0.307 | -0.4148 | No |
| 12 | NUMA1 |  |  | 477 | -0.307 | -0.4104 | No |
| 13 | EPB41L2 |  |  | 488 | -0.310 | -0.4127 | No |
| 14 | TRIO |  |  | 525 | -0.322 | -0.4438 | No |
| 15 | CEP192 |  |  | 531 | -0.324 | -0.4401 | No |
| 16 | CEP57 |  |  | 545 | -0.330 | -0.4452 | No |
| 17 | SASS6 |  |  | 567 | -0.342 | -0.4589 | No |
| 18 | CDK5RAP2 |  |  | 591 | -0.353 | -0.4745 | No |
| 19 | NEK2 |  |  | 628 | -0.374 | -0.5042 | No |
| 20 | SAC3D1 |  |  | 679 | -0.416 | -0.5483 | No |
| 21 | CKAP5 |  |  | 694 | -0.426 | -0.5518 | No |
| 22 | NIN |  |  | 722 | -0.452 | -0.5691 | Yes |
| 23 | TTK |  |  | 724 | -0.454 | -0.5571 | Yes |
| 24 | PCNT |  |  | 725 | -0.454 | -0.5440 | Yes |
| 25 | SMC3 |  |  | 743 | -0.470 | -0.5495 | Yes |
| 26 | KIF5B |  |  | 770 | -0.515 | -0.5638 | Yes |
| 27 | INCENP |  |  | 789 | -0.558 | -0.5680 | Yes |
| 28 | RACGAP1 |  |  | 796 | -0.566 | -0.5583 | Yes |
| 29 | CENPJ |  |  | 799 | -0.568 | -0.5442 | Yes |
| 30 | PLK1 |  |  | 809 | -0.586 | -0.5374 | Yes |
| 31 | LMNB1 |  |  | 818 | -0.606 | -0.5288 | Yes |
| 32 | PIF1 |  |  | 822 | -0.616 | -0.5144 | Yes |
| 33 | AURKA |  |  | 824 | -0.623 | -0.4976 | Yes |
| 34 | BUB1 |  |  | 837 | -0.653 | -0.4922 | Yes |
| 35 | KIF22 |  |  | 842 | -0.678 | -0.4771 | Yes |
| 36 | BIRC5 |  |  | 843 | -0.679 | -0.4575 | Yes |
| 37 | BRCA2 |  |  | 850 | -0.694 | -0.4442 | Yes |
| 38 | ECT2 |  |  | 854 | -0.717 | -0.4268 | Yes |
| 39 | KIF2C |  |  | 855 | -0.721 | -0.4060 | Yes |
| 40 | ANLN |  |  | 860 | -0.733 | -0.3894 | Yes |
| 41 | KIF4A |  |  | 861 | -0.736 | -0.3681 | Yes |
| 42 | FBXO5 |  |  | 864 | -0.742 | -0.3489 | Yes |
| 43 | CCNB2 |  |  | 865 | -0.745 | -0.3274 | Yes |
| 44 | NDC80 |  |  | 869 | -0.762 | -0.3088 | Yes |
| 45 | KIF23 |  |  | 873 | -0.772 | -0.2898 | Yes |
| 46 | KIF20B |  |  | 876 | -0.779 | -0.2696 | Yes |
| 47 | KIF15 |  |  | 890 | -0.834 | -0.2601 | Yes |
| 48 | CENPE |  |  | 899 | -0.873 | -0.2439 | Yes |
| 49 | SMC4 |  |  | 902 | -0.886 | -0.2205 | Yes |
| 50 | KIF11 |  |  | 913 | -0.957 | -0.2041 | Yes |
| 51 | PRC1 |  |  | 919 | -0.980 | -0.1814 | Yes |
| 52 | DLGAP5 |  |  | 923 | -0.998 | -0.1560 | Yes |
| 53 | NUSAP1 |  |  | 929 | -1.112 | -0.1295 | Yes |
| 54 | TPX2 |  |  | 930 | -1.124 | -0.0970 | Yes |
| 55 | CENPF |  |  | 935 | -1.200 | -0.0669 | Yes |
| 56 | CDK1 |  |  | 938 | -1.248 | -0.0331 | Yes |
| 57 | TOP2A |  |  | 942 | -1.417 | 0.0045 | Yes |
Table: GSEA details [plain text format]

  

Fig 2: HALLMARK\_MITOTIC\_SPINDLE: Random ES distribution      
 Gene set null distribution of ES for **HALLMARK\_MITOTIC\_SPINDLE**
